# “Are We Working (Too) Comfortably?”: Understanding the Nature of and Factors Associated with Sedentary Behaviour When Working in the Home Environment

**DOI:** 10.1007/s41542-022-00128-6

**Published:** 2022-11-29

**Authors:** Ailsa Niven, Graham Baker, Eva Coral Almeida, Samantha G Fawkner, Ruth Jepson, Jillian Manner, Sarah Morton, Glenna Nightingale, Divya Sivaramakrishnan, Claire Fitzsimons

**Affiliations:** 1 Physical Activity for Health Research Centre, University of Edinburgh, Edinburgh, Scotland; 2 Scottish Collaboration for Public Health Research & Policy, University of Edinburgh, Edinburgh, Scotland

**Keywords:** Sitting behaviour, Intervention development, Occupational

## Abstract

Home working has increased due to COVID-19, but little is known about how this change has impacted the health risk behaviour of elevated sedentary time. The aim of this cross-sectional exploratory study was to assess occupational sitting behaviour when working at home, and use the Capability Opportunity Motivation-Behaviour (COM-B) model to identify influences on this behaviour. University staff (n = 267; 69% female; 92% white) who were predominantly working from home completed a questionnaire to assess sitting time, sitting breaks, demographic and occupational characteristics, and a 7-item COM-B questionnaire and open-ended questions to assess influences on time spent sitting whilst working from home. Data were analysed descriptively, a repeated measures ANOVA was used to determine differences in the COM-B items, and binary logistic regression was used to examine predictors of sitting time. Staff spent on average 89.5% (SD = 17.1) of their time sitting whilst working at home, and took an average of 1.36 (1.38) sitting breaks per hour. There were significant and meaningful differences in the influence of the COM factors on ability and willingness to reduce sitting behaviour (p < .0001; η_p_^2^ = .38), and the open-ended responses added further context. The included variables accounted for 20.7% of variance in sitting behaviour, with age, sitting breaks, motivation-automatic, and opportunity-physical contributing significantly. Working from home leads to elevated levels of sitting, and the COM-B provides a useful model to identify key influences on ability and willingness to reduce sitting. Strategies incorporating regular breaks, habit formation/reversal, and restructuring the physical environment may be beneficial.

## Introduction

COVID-19 has permanently changed the way many of us work. For large sectors of the working population, spending all or a portion of the week working from home has become part of the ‘new normal’ working landscape (BBC News, 2022; British Council for Offices, 2020; Pew Research Centre, 2022). Working from home is also known as teleworking, and while there are likely to be positive consequences of this shift in working practices (e.g., reduction in commuting time, opportunities for better work: life balance), there are also potentially unintended adverse health effects (Lunde et al., 2022; Oakman et al., 2020).

One such unintended consequence is an increase in sedentary behaviour, defined as any waking behaviour characterized by an energy expenditure ≤ 1.5 METs while in a sitting, reclining or lying posture (Tremblay et al., 2017). Higher levels of sedentary behaviour are a public health hazard with established adverse physical and mental health consequences. For example, 6 to 8 h of sedentary time a day is associated with increased risk of all-cause and cardiovascular disease mortality, Type 2 diabetes, and obesity (Patterson et al., 2018), and a positive association has been reported between mentally passive sedentary behaviour and depression (Huang et al., 2020). Consequently, the World Health Organisation has recommended that all adults should limit the amount of time they spend being sedentary (World Health Organization, 2020). Sedentary behaviour is different from not being physically active; indeed, individuals can be highly sedentary during the working day and physically active at other times (e.g., evening gym class). However, the physical health risks of being highly sedentary are attenuated only with relatively high levels of physical activity (Dempsey et al., 2020; Ekelund et al., 2016).

The workplace has already been identified as a high risk setting for sedentary behaviour. Studies indicate that office-based employees can spend between 58% (Maes et al., 2020), 78% (Hadgraft et al., 2016; Rosenkranz et al., 2020) and 82% (Parry & Straker, 2013) of their working day sitting; equating to up to 438 min/day (Parry & Straker, 2013). Occupational groups with the highest levels of sedentary behaviour include professional, secretarial and administrative, and customer services groups (Kazi et al., 2019), and these groups are also amongst those most likely to be able to work at home (Office for National Statistics, 2020).

Home working has the potential to further increase workday sedentary behaviour, as employees are likely to sit at screens for longer, have more on-line meetings, no longer actively commute to and from work and meetings, and have fewer occupational and social reasons for leaving their workspace. Indeed, one previous study evaluating the impact of introducing flexible working (i.e., being able to work remotely away from the office) reported an increase in actual and perceived workplace sitting time (Olsen et al., 2018b, c). Additionally, emerging evidence suggests that working from home due to COVID restrictions is associated with increased sedentary behaviour (Ráthonyi et al., 2021). For example, compared with not working at home, working at home was associated with ~ 31 (McDowell et al., 2020) and 110 min (or 24% of working time) (Fukushima et al., 2021) more sedentary time per working day.

Despite this recognised increase in sedentary behaviour while working at home, there has been limited consideration of how best to support employees to reduce and/or break up their sedentary behaviour in this setting. There is now substantial research in a work setting that has identified factors that influence sedentary behaviour (e.g., Ojo et al., 2019) and implemented successful interventions (e.g., Chu et al., 2016). However, this research has been focused on ‘traditional’ highly sedentary workplaces such as offices, which may not be applicable to working at home. Indeed, offices differ considerably from the working at home environment in terms of physical set-up, presence of colleagues, types of work activities, and opportunities to move. There is a dearth of intervention research focusing specifically on supporting people to reduce and/or break up their sedentary behaviour while working from home. An exception is Olsen et al., (2018a) who, based on employee suggestions (Olsen et al., 2018c), implemented an intervention integrating action planning, self-monitoring and social support aiming to reduce sitting while working flexibly between home and office environments. However, the authors reported non-significant increases in sitting while working at home, and non-significant decreases in sitting while working in the office, highlighting the need for further intervention research to identify how best to support employees working at home to reduce their sedentary behaviour.

The Behaviour Change Wheel (BCW; Michie et al., 2014; Michie et al., 2011) is a theory-based framework that provides step-by-step guidance on intervention design. The BCW has been used to inform intervention development in a diverse range of contexts, including for example physical activity (Murtagh et al., 2018), hearing aid use (Barker et al., 2016) and reducing COVID-19 transmission (Michie et al., 2020). In brief, there are three stages of intervention development within the BCW. In the first stage, developers specify the target behaviour and identify the sources that are most influential on that behaviour in that specific context. The factors that influence behaviour are categorised using the COM-B model to identify the role of **C**apability, **O**pportunity, and **M**otivation on **B**ehaviour. Capability relates to having the physical (e.g., strength) and psychological (e.g., behavioural regulation) capability to perform the behaviour. Opportunity relates to being in a physical (e.g., resources) and social (e.g., cultural norms) environment that is conducive to undertaking the behaviour. There must also be sufficient motivation to engage in the behaviour and this can be sub-divided into reflective (i.e., involving planning and evaluation) and automatic (e.g., habit) motivation. From this initial behavioural diagnosis the most dominant influences on behaviour can be identified. In Stage 2 of the BCW, intervention developers can map needed changes to relevant intervention functions (e.g., training) and policy categories (e.g., communication/ marketing). The third and final stage involves specifying the content of the proposed intervention by identifying which behaviour change techniques (BCTs; i.e. “an active component of an intervention designed to change behaviour” (Michie et al., 2014, p. 145) should be integrated to address the specified intervention functions. This BCW framework has been used effectively within research focusing on understanding sitting behaviour through the COM-B theoretical lens, and designing interventions to reduce office-based sedentary behaviour (Macdonald et al., 2018; Munir et al., 2018; Ojo et al., 2019; Ojo, Bailey, Hewson, et al.,^2019^ ). However, this office-based research is unlikely to be directly transferable to the home working environment due to the differences in the two environments, as outlined above. To date there is no BCW research that has examined sedentary behaviour in the home working environment and identified the factors that may influence this behaviour in order to understand how best to intervene.

The overall aim of this observational exploratory study was to increase understanding of the sedentary behaviour of University employees in the working from home environment. We have operationalised sedentary behaviour as sitting behaviour, although we recognise there may be circumstances where some workers are not physically able to change their posture and reduce their sitting. The first objective was to assess current sitting behaviour while working at home, to contribute to the limited research in this area. The second objective was to draw from the Behaviour Change Wheel approach to intervention design (Michie et al., 2011), and specifically use the COM-B to characterise participants’ perceived capability, opportunity, and motivation to reduce sitting while working from home. The third objective was to examine the predictive strength of these COM-B factors, in addition to demographic and work-related factors, on the criterion of sitting behaviour while working from home.

## Method

### Participant Characteristics, Inclusion Criteria, and Sampling

Following institutional ethical approval (ref: AN17022021-1), University employees from one institution were invited to participate in the study if they met the inclusion criteria of being aged 18 or older, working predominantly from home at the time of the survey, and with access to an internet connected device. Multiple recruitment pathways were used including staff newsletters, Microsoft Teams posts, social media, and email distribution networks. Focusing on this population enabled us to recruit both professional and administrative staff. From a potential sampling pool of > 15,000 staff, 332 participants (73% female; 92% White; 82% full-time) completed the study. Recruitment was open from the 13th April 2021 for four weeks. During this period, Scotland moved from COVID-19 Protection Level 4 to Level 3, with The Scottish Government recommending working from home where possible.

### Measures

An online Qualtrics questionnaire was developed, which included seven sections, and the data from three sections (demographics, occupational sitting and breaks from sitting, COM-B variables) are reported in this paper. The remaining four sections related to mental and work well-being, and musculoskeletal health and will be reported elsewhere (The full questionnaire is accessible here).

### Demographic data

Participants reported their age, gender, ethnicity, work role (i.e., type, if full-time/part-time, and percentage of time currently working at home), length of time at the institution, and if they had undertaken generic in-house training regarding setting up a home-work space.

### Occupational Sitting and Breaks from Sitting

The Occupational Sitting and Physical Activity Questionnaire (OSPAQ) (Chau et al., 2012) was used to assess sitting time during working hours. The OSPAQ has acceptable measurement properties for assessing occupational sitting (Maes et al., 2020). Participants were asked to report how many hours and days they worked in the last seven days, and indicate what percentage of the working day was made up of sitting, standing, walking, and heavy labour activities. From this, the percentage of time worked spent sitting was calculated, and total number of minutes spent sitting while working. Participants with missing data for hours, days, or percentages were excluded.

The number of breaks from sitting participants took per hour was also assessed using a measure with acceptable reliability and validity (Sudholz et al., 2018): “*In the last 7 days, how many breaks from sitting did you take (on average) per hour, while at work? This could include standing, stretching, taking a short walk. Please do not count lunch breaks or tea breaks*”. The text ‘on average’ was added to further direct the respondents. Sudholz et al., (2018) advise that scoring should be truncated at six breaks/hour. However, for our data if it was evident that some participants (n = 18 out of 267 participants) had reported breaks per day (e.g., n = 20 breaks and/or including if they specified ‘per day’), then this was divided by 7.5, which is the standard working day, to equate to per hour.

### COM-B Beliefs

Perceived capability, opportunity and motivation to reduce sitting while working from home, were assessed using an adapted form of the 6-item questionnaire developed by Keyworth et al., (2020). Participants were asked to rate on an eleven-point scale (0 strongly disagree to 10 strongly agree) their ability and willingness to reduce the time they spend sitting while working at home in relation to capability, opportunity and motivation. For example, ‘*I have the physical opportunity to reduce the time I spend sitting whilst working from home (e.g., sufficient time, equipment, space, reminders)*.’ Consistent with Spence et al., (2021), we differentiated between psychological capability (i.e., having the ability to engage in appropriate memory, attention and decision-making processes) and knowledge, and thus added an additional item relating to knowledge. Therefore, the COM-B measure used in this study included seven predictor variables, with a single item assessing each of the variables of physical capability, psychological capability, social opportunity, physical opportunity, automatic motivation, reflective motivation, and knowledge. Participants were also invited to provide any open-ended additional comments relating to their ability and willingness to reduce their time spent sitting while working from home.

### Data Collection

Following invitation to participate in the study, interested participants could ‘click through’ to the online Qualtrics questionnaire. The Participant Information Sheet was integrated into the questionnaire and provided full details of the study. In order to complete the questionnaire, participants were required to indicate their granular consent to participate in the study by actively ‘ticking’ a series of consent statements. Unless consent was provided, participants could not progress to the questionnaire. Participants were able to progress through the questionnaire responding only to those questions they chose to (i.e., there were no force response questions). Participants’ responses were included in the study only if they actively submitted the questionnaire by clicking on a ‘submit’ button. Participants were incentivised to participate through the offer of being entered into a prize draw to win one of five £50 vouchers.

### Analysis

The data were cleaned for missing data and unintelligible data entries. The OSPAQ and sitting break data were initially analysed descriptively to determine the extent of sitting and breaks while working at home. Participant’s ability and willingness to reduce the time they spend sitting in relation to capability, opportunity, and motivation was analysed using a repeated measures ANOVA with post-hoc paired sample t-tests (with Bonferroni correction for 21 multiple comparisons; p < .0024) and Hedge’s g calculations to determine the significance and size of differences between these variables.

The open-ended responses in relation to factors influencing ability and willingness to reduce time spent sitting were imported into NVivo. Each individual response was initially deductively analysed by one researcher (AN) into categories aligning with physical and psychological capability, physical and social opportunity, and reflective and automatic motivation (Michie et al., 2011). Within each of these categories, the data were then inductively analysed to cluster together similar comments into sub-themes. Comments that did not fit these pre-defined categories were also inductively analysed by AN to identify additional relevant categories. The other co-authors reviewed the analysis.

A parsimonious logistic regression was constructed to quantify the impact of key predictive variables on the criterion variable of workplace sitting time above and below the OSPAQ mean. A linear regression where sitting time was treated as a continuous variable was not feasible as the data were not normally distributed. In the absence of a standardised threshold for high sitting time at work, we created two categories representing equal to and above the mean, and below the mean. Included variables were treated as either categorical or continuous based on question format, and as detailed in Table 1. Data on sitting breaks were visually inspected to determine an appropriate mid-point cut-off and the categories of ‘few’ (< 2) and ‘many’ (≥ 2) were created.

**Table 1:** Descriptive results for participant characteristics, and sitting behaviour

Variables (n = 267)	Frequency (%)	Mean (SD)
Age		
18–30	27 (10%)	-
31–40	80 (30%)	-
41–50	89 (33%)	-
51–60	57 (21%)	-
61+	14 (5%)	-
Gender^a^		
female	184 (69%)	-
Male	80 (30%)	-
Ethnicity		
White	246 (92%)	-
non-white^b^	18 (7%)	-
Job role		
Professional services	194 (73%)	-
Academic	61 (23%)	-
Technician	4 (1.5%)	-
Other	8 (3%)	-
Time worked at institution		
Less than 1 year	22 (8%)	-
1–10 years	160 (60%)	-
11–20 years	50 (19%)	-
20 + years	35 (13%)	-
Undertook training		
Yes	147 (55%)	-
No/ Unaware	120 (45%)	-
Sitting behaviour		
% of workday spent sitting	-	89.53 (17.14)
Minutes spent sitting at work	-	397.29 (99.11)
Sitting breaks		
Number of breaks per hour	-	1.36 (1.38)
<2 breaks per hour	195 (73%)	
≥2 breaks per hour	72 (27%)	

^a^Responses included non-binary (n = 1) and prefer not to say (n = 2), but due to the small size of these groups they were not included in the analyses. ^b^Responses included five specified ethnic groups collapsed to one non-white group for analysis due to size, and prefer not to say (n = 3) excluded from analysis due to size

## Results

A full data set was available for 267 participants, who worked mainly full-time (81%) and were working predominantly at home (mean = 95.4%; SD = 14.5% of the working week). Table 1 presents the descriptive data for the participant characteristics and sitting behaviour. Participants represented a range of ages, were predominantly female (69%), white (92%), working in a professional services role (73%), and the majority (60%) had spent between 1 and 10 years at the institution. There was a near even split between those who had undertaken in-house training on working at home, and those who had not. Staff self-reported spending on average 89.5% (SD = 17.1%) of their working day sitting. This equated to an average of 397 (99) minutes per day. Participants reported taking an average of 1.36 (SD = 1.38) breaks per hour (median = 1; IQ Range = 1.5), and most participants reported taking less than two breaks an hour (n = 195), with the remainder reporting two or more breaks per hour (n = 72). Table 2 reports a comparison of the mean (SD) data for the variables assessing capability, opportunity and motivation. There were significant and meaningful differences in participant’s rated ability and willingness to reduce their time spent sitting in relation to physical capability, psychological capability, social opportunity, physical opportunity, automatic motivation, reflective motivation and knowledge (p < .0001; η_p_^2^ = 0.38). There were significant differences between all variables with the exception of three paired comparisons where the p value exceeded the Bonferroni adjusted 0.0024. Specifically, there was no significant difference between social opportunity and physical opportunity (p = .009), psychological capability and reflective motivation (p = .009), and psychological capability and knowledge (p = .095). Effect sizes ranged from small (e.g., physical opportunity vs. reflective motivation) to very large (e.g., physical capability vs. automatic motivation). Participants perceived that they were least able to automatically reduce their sitting behaviour, and that they scored lower relative to the other variables on social and physical opportunity to reduce their sitting. Relative to the other variables, participants scored highest on perceived physical capability and knowledge. Notably, there were large standard deviations for several variables suggesting a variation in participants’ responses.

**Table 2:** Comparison of the mean and standard deviation of each COM variable

COM Variable	Mean (SD)
Capability – physical	8.76 (1.93)^a^
Capability – psychological	6.93 (2.56)^b^
Opportunity – physical	5.64 (3.19)^c^
Opportunity – social	5.16 (3.11)^a^
Motivation – reflective	6.46 (2.61)^a^
Motivation – automatic	3.17 (2.80)^a^
Knowledge	7.19 (2.54)^a^

^a^significantly different from all other variables; ^b^significantly different from all variables with the exception of reflective motivation and knowledge; ^c^significantly different from all variables with the exception of social opportunity

Table 3 presents the analysis of the open-ended responses (n = 101) relating to participants’ ability and willingness to reduce their time spent sitting while working from home. The majority of these comments were clustered into the categories of capability, opportunity and motivation. There were limited comments regarding physical capability, but it was evident that psychological capability was perceived to be influential on behaviour. The most common psychological capability theme related to the ability to reduce sitting being limited because participants were immersed in their work. For example, “*It’s hard to remember to move when you are deep in marking or writing a new course. I feel I will lose the thread and have to start again*”.

**Table 3:** Analysis of open-ended response relating to ability and willingness to reduce sitting while working at home

Theme	Sub-Theme
Capability	
Psychological (n = 16)	Become immersed in work (n = 10)
	Lack of knowledge or belief (n = 3)
	Awareness of the benefits (n = 2)
	Reduce sitting when remember (n = 1)
Physical (n = 4)	Ongoing health issue (n = 3)
	Inability to physically complete (n = 1)
Opportunity	
Lack of Physical (n = 68)	Work demands reduce opportunities to reduce sitting (n = 33)
	Lack of standing desk or appropriate space (n = 28)
	Home office less conducive to reducing sitting than work office (n = 4)
	Time pressure from non-work responsibilities (n = 2)
	Impact of weather (n = 1)
Presence of physical (n = 10)	Having a standing desk (n = 5)
	Using activity monitor (n = 3)
	Influenced by external campaign (n = 1)
	Non-work activities reduce sitting (n = 1)
Social (n = 9)	Social pressure (n = 8)
	Lack of organisational support (n = 1)
Motivation	
Reflective (n = 21)	Beliefs about negative consequences on work productivity (n = 9)
	Intention to reduce sitting (n = 6)
	Lack of intention to reduce sitting (n = 5)
	Reduced belief in capability to reduce sitting (n = 1)
Automatic (n = 2)	Emotion driven (n = 1)
	Habit (n = 1)
Other	
Physically active	Physically active at other times to compensate (n = 5)

The lack of physical opportunity was the most commonly reported theme impacting on ability and willingness to reduce sitting. Specifically, work demands were perceived as a barrier, for example one participant noted “*I’m so busy that I only leave my desk to make a coffee or eat*”. Further, lack of space, or not having a standing desk were also commonly reported barriers, with one participant stating “*no space at home, limited furniture/equipment available. I work from my dining table in a small flat*”. Conversely, having a standing desk facilitated reduced sitting, and some participants reported using an activity monitor to remind them to stand. There were some comments relating to social opportunity, with several participants noting that there was social pressure to be at their desk, thus limiting the opportunity to reduce sitting. For example, “*It does not always feel acceptable to be away from my desk during working hours. This may be perceived or real”.*

Reflective motivation was the second most commonly reported theme, with participants flagging concerns about the consequences of reducing their sitting on work productivity. For example, “*Not so much a problem with my ability or willingness, it’s just I can’t do a lot of my job while standing and if I take breaks to move I’ll have to work later to make up for it”*. Some participants reported an intention to reduce sitting, but other participants indicated that they had no intention to do so. There were only two comments relating to automatic motivation, reflecting how low mood and habitual sitting negatively impacted on ability to reduce sitting behaviour.

A final theme was identified that included comments from participants that they chose to be physically active at other times of the day to compensate for their lack of movement during the working day. For example, one participant reported “*I use the time I’m not working/traveling to work to be more active”*.

Table 4 presents the findings from the logistic regression. Overall, the predictive variables accounted for 20.7% of the variance in the criterion variable of sitting time while working from home. There was limited influence of demographic variables on sitting time, with the exception of age where there was increased odds of greater sitting in age groups 31–40 and 51–60 compared with 18–30 group. Sitting time was not related to job role, nor time spent at the institution, nor whether participants had undertaken training on working from home. Two of the COM-B factors were significantly related to sitting time. Specifically, for both automatic motivation and physical opportunity, an increased score of one unit on the scale was significantly associated with a reduction in being in the highest category of sitting, by 19% and 16%, respectively. Finally, having many breaks (i.e., ≥ 2) reduced the odds of being in the highest sitting category by 53% compared with having few breaks (i.e., < 2).

**Table 4:** Odds ratio and 95% confidence interval for above and below the mean of sitting behaviour when working at home for demographic, work-related, sitting breaks, and COM-B variables

Predictive variables	Odds Ratio	95% Confidence Interval	p-value
Age			
18–30	REF	REF	
31–40	3.02	1.11, 8.45	0.031*
41–50	2.20	0.80, 6.19	0.13
51–60	7.03	2.20, 24.0	0.001*
61+	3.41	0.70, 17.6	0.13
Gender			
female	REF	REF	
male	1.15	0.61, 2.21	0.7
Ethnicity			
white	REF	REF	
non-white	1.92	0.62, 6.72	0.3
Job role			
Professional services	REF	REF	
Academic	0.73	0.35, 1.52	0.4
Technician	0.63	0.06, 6.67	0.7
Other	1.50	0.29, 9.30	0.6
Time worked at institution			
Less than 1 year	REF	REF	
1–10 years	2.09	0.74, 5.96	0.2
11–20 years	1.37	0.41, 4.62	0.6
20 + years	0.89	0.23, 3.40	0.9
Undertook training			
Yes	REF	REF	
No/ Unaware	0.80	0.43, 1.50	0.5
Sitting breaks			
Few (< 2) (n = 195)	REF	REF	
Many (≥ 2) (n = 72)	0.46	0.24, 0.88	0.019*
COM-B			
Capability – physical	1.04	0.87, 1.23	0.7
Capability – psychological	1.00	0.86, 1.15	> 0.9
Opportunity – physical	0.84	0.74, 0.95	0.005*
Opportunity – social	1.03	0.92, 1.16	0.6
Motivation – reflective	1.04	0.91, 1.19	0.6
Motivation – automatic	0.80	0.71, 0.91	< 0.001*
Knowledge	1.00	0.85, 1.16	> 0.9

Note R^2^ = 0.207;

*significant p < .05

## Discussion

The working landscape has changed as a consequence of COVID-19, with many sectors looking to retain a working pattern whereby employees spend at least a proportion of the week working at home. This is the first study to conduct a detailed theoretically-informed behavioural analysis of sedentary behaviour in employees who are working from home. This study provides insight into the levels of sitting while working at home, and identifies factors that influence this behaviour. These findings can be used to inform interventions designed to offset potential adverse physical and mental health consequences from this shift in working practice.

Participants reported spending an average of 89.5% of their working week sitting, and that equated to nearly 400 min. Comparison with data from an office environment using the same measure (i.e., OSPAQ) suggests that employees working at home are likely to sit more. Rosenkranz et al., (2020) reported an average of 78.1% (SD = 19) in office-based workers (n = 2068), and Maes et al., (2020) reported 58% (SD = 33) in sedentary job-types (n = 65). The suggestion that sitting time is elevated while working at home is consistent with previous research on flexible working contrasting the home with office environments (Olsen et al., 2018b, c), and studies that have reported increased sedentary behaviour in workers at home during COVID lockdown (Fukushima et al., 2021; McDowell et al., 2020; Ráthonyi et al., 2021). Participants reported taking an average of 1.36 breaks per hour. Although there is limited literature using the same sitting breaks measure, the data are similar to the original validation study that collected data in office-based workers (Sudholz et al., 2018) (i.e., median = 1 in each study).

Given that working at home appears to increase sitting time, it is important to understand what factors may influence this behaviour in order to design appropriate interventions. The COM-B (Michie et al., 2011) provides a useful theoretical framework to advance understanding on modifiable factors influencing sitting when working at home, and inform potential intervention strategies. The potential influence of capability, opportunity and motivation are discussed in turn. Further, consideration of the findings of this study in comparison with office-based studies informed by the COM-B (i.e., Macdonald et al., 2018; Munir et al., 2018; Ojo, Bailey, Brierley, et al., 2019) enables identification of similar and different factors influencing sitting behaviour in the home working environment.

It was evident that participants in this study perceived there were differences in the extent to which capability, opportunity and motivation influenced their ability and willingness to reduce their sitting. Relative to the other COM-B factors, participants in this study perceived high levels of both physical and psychological capability, and knowledge to reduce their sitting; and this was also reflected in the qualitative data where there were limited comments on these factors. An exception was that several participants highlighted they can become immersed in their work, and therefore their psychological capability to limit sitting is reduced. These findings are somewhat consistent with office-based studies. Specifically, previous research also highlights that physical capability was not perceived as a barrier to reducing sitting in the office (Macdonald et al., 2018; Munir et al., 2018; Ojo, Bailey, Hewson, et al., 2019). Further, it was recognised previously that psychological capability can inhibit the behaviour when participants forget to reduce sitting due to being absorbed in their work (Munir et al., 2018; Ojo, Bailey, Hewson, et al., 2019). Previous research has suggested introducing prompts (e.g., calendar reminders/ alarms) to disrupt intense concentration and as a reminder to enact the intention to reduce sitting (Ojo, Bailey, Brierley, et al., 2019), and such strategies could be transferable to the working at home environment. Contrary to the findings of this study, in previous office-based studies participants reported limited knowledge and capability to self-regulate their sitting behaviour (Macdonald et al., 2018; Munir et al., 2018; Ojo, Bailey, Brierley, et al., 2019). It may be that participants in this study have been exposed to more information on sitting behaviour, but nevertheless additional research would be valuable to understand further the knowledge levels of workers at home regarding why and how to break up sitting. Such research could inform whether education-focused interventions are needed in this setting.

Relative to other factors, physical opportunities to reduce sitting were perceived by participants, on average, to be limited in this study. These limitations included both the lack of availability of space and standing desks in the home environment, and workload demands. Other studies have also reported on the importance of opportunities in the physical office environment to reduce sitting through movement around multi-floor buildings (Macdonald et al., 2018), by having centralised printers and waste bins, and the implementation of height-adjustable desks (Munir et al., 2018; Ojo, Bailey, Brierley, et al., 2019). These findings are consistent between the different work environments, and some solutions, such as the introduction of a height-adjustable desk, could be financially and logistically feasible for some organisations and homes, but not for others. It is likely that the unique working from home environment will require alternative solutions to the office in order to maximise the workspace available, and further research is needed to inform these strategies. Other studies have also recognised that work demands can negatively impact on the opportunity to reduce sitting (Macdonald et al., 2018; Ojo, Bailey, Brierley, et al., 2019), and finding strategies to address this perception should be a key focus of future research.

In relation to social opportunities, the findings of this study indicate that participants perceived, on average, these were limited, potentially influenced by concerns that others in the work environment would expect participants to be sitting at their desk. This finding is consistent with office-based studies that have highlighted the influence of being judged by others, and social norms on sitting behaviour (Macdonald et al., 2018; Munir et al., 2018; Ojo, Bailey, Brierley, et al., 2019). Although when working at home there is an absence of colleagues being physically present, there is some suggestion that the digital environment can still create space where participants feel observed and monitored, and this may impact on the opportunity to reduce sitting. Strategies such as implementing social support for the behaviour and modelling of the behaviour from both senior staff and colleagues have been suggested previously (Munir et al., 2018), and could also work well in digital interactions.

Reflective motivation was the factor that scored third highest in this study, suggesting that participants were motivated to reduce their sitting. However, the qualitative responses provided some further insight, with participants reporting beliefs that reducing their sitting would negatively impact their work productivity, thus potentially reducing motivation. Office-based studies have also reported this concern (Macdonald et al., 2018), but other participants have recognised that reducing sitting could enhance their productivity (Ojo, Bailey, Hewson, et al., 2019). There is mixed evidence on the relationship between sedentary behaviour and productivity, but a recent large-scale study suggesting there was no relationship (Rosenkranz et al., 2020), and reviews of sedentary interventions indicate limited effect on productivity (e.g., Ojo et al., 2018). These findings could be used to educate employees as to the limited effect of reducing sitting time on productivity, thus challenging beliefs regarding negative consequences, which may help motivate employees to reduce their sitting at home or in the office.

The quantitative data indicated that participants had low levels of automatic motivation to reduce their sitting, suggesting that this behaviour was not habitually executed (i.e., with low levels of consciousness). This may suggest that sitting was habitual for these employees, which is consistent with previous research in the office environment (Macdonald et al., 2018; Munir et al., 2018; Ojo, Bailey, Hewson, et al., 2019). Given that sitting behaviour can be highly habitual, it is not surprising that the opposite behaviour (i.e., breaking up sitting) is not automatically undertaken. Further evidence for the important role of habit in understanding sitting behaviour was reported by Howlett et al. (2021) where habit was identified as the strongest predictor of weekly sitting in a study examining influences of variables from the COM-B and Theory of Planned Behaviour. Future research could incorporate principles of both habit formation and habit breaking in order to address both aspects of increasing strategies to break up sitting and reduce prolonged sitting, respectively (Gardner et al., 2020); and this also reflects recommendations within the office environment (Munir et al., 2018; Ojo, Bailey, Brierley, et al., 2019).

Collectively, the variables in this study accounted for over 20% of the variance in sitting behaviour, which is a meaningful contribution and provides important insight into factors influencing sitting behaviour at home. There was limited influence of demographic characteristics on sitting behaviour, with the exception of age where being older than 18–30 was related to increased sitting in two of the older age categories. This finding is consistent with previous research showing increased sitting with age compared with younger groups (Strain et al., 2018). It was unexpected that this finding was not evident across all older age groups. The lack of significant finding for the 61 + group may be due to low statistical power attributed to small sample size; however it was unexpected that being in the 41–50 group was not associated with increased sitting. Further research is needed to ‘unpack’ the influence of age on sitting time while working at home, as it is likely that contextual influences, such as domestic demands, may impact on behaviour. Sitting behaviour was not associated with employment related characteristics such as job type, time at the institution, nor whether employees had undertaken specific health and safety training related to working at home. Unsurprisingly, having breaks from sitting was an important behaviour associated with reduced sitting time, highlighting the importance of encouraging regular breaks to stand, stretch and take a short walk. Two COM-B factors made a significant contribution. Specifically, enhanced automatic motivation and physical opportunity were significantly associated with lower levels of sitting in this study. Both of these variables were amongst the lowest scored COM-B factors, suggesting they should be a priority for intervention in this population.

In summary, the model accounted for more than 20% of variance in self-reported daily sitting behaviour, with age, number of breaks, and automatic motivation and physical opportunity all making significant and meaningful contributions. It is notable that this finding is less than the 27% variance in sitting behaviour across a week accounted for by the COM-B model reported by Howlett et al., (2021), perhaps suggesting other variables may also be important in the working at home environment, or that the COM-B is a better predictor of weekly rather than daily sitting. There is clearly a need for further research to examine further the influences on sitting behaviour in this specific context, and further evaluate the value of the COM-B model. For example, in addition to self-report data future research could also incorporate an objective assessment of the organisational characteristics and work demands.

While this study has identified factors associated with sitting time, the cross-sectional nature of this study is a limitation to drawing strong conclusions relating to the influence of these factors. Future longitudinal and intervention research would be valuable to explore the predictive value of different factors on behaviour. An additional limitation of this study is a focus on a single organisation and population, albeit with varied job roles. Further, although the sample size was substantial and multiple recruitment strategies were used, the recruited participants represented only a small percentage of the potential participant pool and therefore may not be representative. Future research should aim to recruit a larger sample. A larger sample would also allow an analysis strategy that could provide a more nuanced insight into the predictors of sitting time when home working by considering this variable on a continuous scale rather than as a dichotomous variable. The reliance on self-report measures of sitting behaviour is a further limitation of this study. Although the OSPAQ and SITBRQ have acceptable reliability and validity for assessing sitting while working (Maes et al., 2020; Sudholz et al., 2018), and were especially appropriate and practical for survey-level data during lockdown restrictions, device-based measures would have enhanced the quality of the behavioural data. Finally, it is increasingly recognised that a focus on only sedentary behaviour can be a limitation in fully understanding the health consequences of this behaviour. In future research, consideration should be given to how the time spent sitting when working can influence movement/non-movement patterns over a 24-hour period, with the ultimate goal of a healthy 24-hour composition in relation to sedentary behaviour, physical activity, and also sleep (Rollo et al., 2020).

## Conclusion

This research makes an important first contribution to understanding the nature of and factors associated with sitting behaviour in the working at home environment. There is growing evidence that working at home results in greater sitting time than working in an office environment. The COM-B model provides a useful framework for understanding influences on sitting behaviour when working at home, and identifying targets for interventions. Based on this study, priorities should be to enhance automatic motivation and physical opportunity influences and encourage regular breaks. Nevertheless, there is clearly a need for further studies with additional occupational sectors to build on this evidence to inform future interventions to support employees working at home.

## Data Availability

The data that support the findings of this study are openly available in datashare.ed.ac.uk – 10.7488/ds/3402

## References

[CR1] Barker F, Atkins L, de Lusignan S (2016). Applying the COM-B behaviour model and behaviour change wheel to develop an intervention to improve hearing-aid use in adult auditory rehabilitation. International Journal of Audiology.

[CR2] BBC News (2022). Hybrid working is here to stay, say managers. Retrieved from https://www.bbc.co.uk/news/business-60421056

[CR3] British Council for Offices (2020). 5 October 2020). Majority of workers plan a return to the office, but home working here to stay. Retrieved from http://www.bco.org.uk/News/News46982.aspx

[CR4] Chau JY, Van Der Ploeg HP, Dunn S, Kurko J, Bauman AE (2012). Validity of the occupational sitting and physical activity questionnaire. Medicine And Science In Sports And Exercise.

[CR5] Chu AHY, Ng SHX, Tan CS, Win AM, Koh D, Müller-Riemenschneider F (2016). A systematic review and meta-analysis of workplace intervention strategies to reduce sedentary time in white-collar workers. Obesity Reviews.

[CR6] Dempsey PC, Biddle SJH, Buman MP, Chastin S, Ekelund U, Friedenreich CM, Bull F (2020). New global guidelines on sedentary behaviour and health for adults: broadening the behavioural targets. Int J Behav Nutr Phys Act.

[CR7] Ekelund U, Steene-Johannessen J, Brown WJ, Fagerland MW, Owen N, Powell KE, Lee IM (2016). Does physical activity attenuate, or even eliminate, the detrimental association of sitting time with mortality? A harmonised meta-analysis of data from more than 1 million men and women. Lancet.

[CR8] Fukushima N, Machida M, Kikuchi H, Amagasa S, Hayashi T, Odagiri Y, Inoue S (2021). Associations of working from home with occupational physical activity and sedentary behavior under the COVID-19 pandemic. Journal of Occupational Health.

[CR9] Gardner B, Rebar AL, Lally P, Hamilton K, Cameron LD, Hagger MS, Hankonen N, Lintunen T (2020). Habit Interventions. The Handbook of Behavior Change.

[CR10] Howlett N, Schulz J, Trivedi D, Troop N, Chater A (2021). Determinants of weekly sitting time: construct validation of an initial COM-B model and comparison of its predictive validity with the Theory of Planned Behaviour. Psychology & Health.

[CR11] Huang Y, Li L, Gan Y, Wang C, Jiang H, Cao S, Lu Z (2020). Sedentary behaviors and risk of depression: a meta-analysis of prospective studies. Translational Psychiatry.

[CR12] Kazi A, Haslam C, Duncan M, Clemes S, Twumasi R (2019). Sedentary behaviour and health at work: an investigation of industrial sector, job role, gender and geographical differences. Ergonomics.

[CR13] Keyworth C, Epton T, Goldthorpe J, Calam R, Armitage CJ (2020). Acceptability, reliability, and validity of a brief measure of capabilities, opportunities, and motivations (“COM-B”). British Journal of Health Psychology.

[CR14] Lunde LK, Flovik L, Christensen JO, Johannessen HA, Finne LB, Jorgensen IL, Vleeshouwers J (2022). The relationship between telework from home and employee health: a systematic review. Bmc Public Health.

[CR15] Macdonald, B. W., Fitzsimons, C., & Niven, A. (2018). Using the COM-B model of behaviour to understand sitting behaviour in U.K. office workers. *Sport and Exercise Psychology Review, 14, *23-32.

[CR16] Maes I, Ketels M, Van Dyck D, Clays E (2020). The occupational sitting and physical activity questionnaire (OSPAQ): a validation study with accelerometer-assessed measures. Bmc Public Health.

[CR17] McDowell CP, Herring MP, Lansing J, Brower C, Meyer JD (2020). Working From Home and Job Loss Due to the COVID-19 Pandemic Are Associated With Greater Time in Sedentary Behaviors. Front Public Health.

[CR18] Michie S, Atkins L, West R (2014). *The Behaviour Change Wheel: A Guide to Designing Interventions*.

[CR19] Michie S, van Stralen MM, West R (2011). The behaviour change wheel: A new method for characterising and designing behaviour change interventions. Implementation Science.

[CR20] Michie S, West R, Rogers MB, Bonell C, Rubin GJ, Amlôt R (2020). Reducing SARS-CoV-2 transmission in the UK: A behavioural science approach to identifying options for increasing adherence to social distancing and shielding vulnerable people. The British Journal Of Health Psychology.

[CR21] Munir F, Biddle SJH, Davies MJ, Dunstan D, Esliger D, Gray LJ, Edwardson CL (2018). Stand More AT Work (SMArT Work): using the behaviour change wheel to develop an intervention to reduce sitting time in the workplace. Bmc Public Health.

[CR22] Murtagh EM, Barnes AT, McMullen J, Morgan PJ (2018). Mothers and teenage daughters walking to health: using the behaviour change wheel to develop an intervention to improve adolescent girls’ physical activity. Public Health.

[CR23] Oakman J, Kinsman N, Stuckey R, Graham M, Weale V (2020). A rapid review of mental and physical health effects of working at home: how do we optimise health?. Bmc Public Health.

[CR24] Office for National Statistics (2020). Which jobs can be done from home? Retrieved from https://www.ons.gov.uk/employmentandlabourmarket/peopleinwork/employmentandemployeetypes/articles/whichjobscanbedonefromhome/2020-07-21

[CR25] Ojo SO, Bailey DP, Brierley ML, Hewson DJ, Chater AM (2019). Breaking barriers: using the behavior change wheel to develop a tailored intervention to overcome workplace inhibitors to breaking up sitting time. Bmc Public Health.

[CR26] Ojo SO, Bailey DP, Chater AM, Hewson DJ (2018). The Impact of Active Workstations on Workplace Productivity and Performance: A Systematic Review. International Journal Of Environmental Research And Public Health.

[CR27] Ojo, S. O., Bailey, D. P., Hewson, D. J., & Chater, A. M. (2019). Perceived barriers and facilitators to breaking up sitting time among desk-based office workers: A qualitative investigation using the TDF and COM-B. *International Journal of Environmental Research And Public Health*, *16*(16), doi:10.3390/ijerph16162903 10.3390/ijerph16162903 PMC6720704 31416112

[CR28] Olsen HM, Brown WJ, Kolbe-Alexander T, Burton NW (2018). A Brief Self-Directed Intervention to Reduce Office Employees’ Sedentary Behavior in a Flexible Workplace. Journal Of Occupational And Environmental Medicine.

[CR29] Olsen HM, Brown WJ, Kolbe-Alexander T, Burton NW (2018). Flexible Work: The Impact of a New Policy on Employees’ Sedentary Behavior and Physical Activity. Journal Of Occupational And Environmental Medicine.

[CR30] Olsen HM, Brown WJ, Kolbe-Alexander T, Burton NW (2018). Physical activity and sedentary behaviour in a flexible office-based workplace: Employee perceptions and priorities for change. Health Promot J Austr.

[CR31] Parry S, Straker L (2013). The contribution of office work to sedentary behaviour associated risk. Bmc Public Health.

[CR32] Patterson R, McNamara E, Tainio M, de Sá TH, Smith AD, Sharp SJ, Wijndaele K (2018). Sedentary behaviour and risk of all-cause, cardiovascular and cancer mortality, and incident type 2 diabetes: a systematic review and dose response meta-analysis. European Journal of Epidemiology.

[CR33] Pew Research Centre (2022). COVID-19 Pandemic Continues To Reshape Work in America. Retrieved from https://www.pewresearch.org/social-trends/2022/02/16/covid-19-pandemic-continues-to-reshape-work-in-america/

[CR34] Ráthonyi, G., Kósa, K., Bács, Z., Ráthonyi-Ódor, K., Füzesi, I., Lengyel, P., & Bácsné Bába, É. (2021). Changes in workers’ physical activity and sedentary behavior during the COVID-19 Pandemic. *Sustainability, 13*(17), 9524. https://doi.org/10.3390/su13179524

[CR35] Rollo S, Antsygina O, Tremblay MS (2020). The whole day matters: Understanding 24-hour movement guideline adherence and relationships with health indicators across the lifespan. J Sport Health Sci.

[CR36] Rosenkranz, S. K., Mailey, E. L., Umansky, E., Rosenkranz, R. R., & Ablah, E. (2020). Workplace sedentary behavior and productivity: A cross-sectional study. *International Journal Of Environmental Research And Public Health*, *17*(18), doi:10.3390/ijerph17186535 10.3390/ijerph17186535 PMC7558581 32911740

[CR37] Spence JC, Rhodes RE, McCurdy A, Mangan A, Hopkins D, Mummery WK (2021). Determinants of physical activity among adults in the United Kingdom during the COVID-19 pandemic: The DUK-COVID study. The British Journal Of Health Psychology.

[CR38] Strain T, Kelly P, Mutrie N, Fitzsimons C (2018). Differences by age and sex in the sedentary time of adults in Scotland. Journal Of Sports Sciences.

[CR39] Sudholz B, Ridgers ND, Mussap A, Bennie J, Timperio A, Salmon J (2018). Reliability and validity of self-reported sitting and breaks from sitting in the workplace. Journal of Science and Medicine in Sport.

[CR40] Tremblay MS, Aubert S, Barnes JD, Saunders TJ, Carson V, Latimer-Cheung AE, Chinapaw MJM (2017). Sedentary Behavior Research Network (SBRN) - Terminology Consensus Project process and outcome. Int J Behav Nutr Phys Act.

[CR41] World Health Organization (2020). *WHO guidelines on physical activity and sedentary behaviour*. Retrieved from https://apps.who.int/iris/rest/bitstreams/1315866/retrieve 10.1136/bjsports-2020-102955 PMC7719906 33239350

